# Refinement of the Fusion Tag PagP for Effective Formation of Inclusion Bodies in Escherichia coli

**DOI:** 10.1128/spectrum.03803-22

**Published:** 2023-05-24

**Authors:** Xuefeng Li, Baorong Zhang, Quan Hu, Changchao Chen, Jiahua Huang, Lu Liu, Shengbin Wang

**Affiliations:** a College of Life Sciences, South China Agricultural University, Guangzhou, People’s Republic of China; b Guangdong Provincial Key Laboratory of Protein Function and Regulation in Agricultural Organisms, Guangzhou, People’s Republic of China; Suranaree University of Technology

**Keywords:** fusion expression, inclusion body, PagP, target protein recovery, antimicrobial peptides

## Abstract

Methods for efficient insoluble protein production require further exploration. PagP, an Escherichia coli outer membrane protein with high β-sheet content, could function as an efficient fusion partner for inclusion body-targeted expression of recombinant peptides. The primary structure of a given polypeptide determines to a large extent its propensity to aggregate. Herein, aggregation “hot spots” (HSs) in PagP were analyzed using the web-based software AGGRESCAN, leading to identification of a C-terminal region harboring numerous HSs. Moreover, a proline-rich region was found in the β-strands. Substitution of these prolines by residues with high β-sheet propensity and hydrophobicity significantly improved its ability to form aggregates. Consequently, the absolute yields of recombinant antimicrobial peptides Magainin II, Metchnikowin, and Andropin were increased significantly when expressed in fusion with this refined version of PagP. We describe separation of recombinant target proteins expressed in inclusion bodies fused with the tag. An artificial NHT linker peptide with three motifs was implemented for separation and purification of authentic recombinant antimicrobial peptides.

**IMPORTANCE** Fusion tag-induced formation of inclusion bodies provides a powerful means to express unstructured or toxic proteins. For a given fusion tag, how to enhance the formation of inclusion bodies remains to be explored. Our study illustrated that the aggregation HSs in a fusion tag played important roles in mediating its insoluble expression. Efficient production of inclusion bodies could also be implemented by refining its primary structure to form a more stable β-sheet with higher hydrophobicity. This study provides a promising method for improvement of the insoluble expression of recombinant proteins.

## INTRODUCTION

Diverse conditions can alter protein homeostasis, resulting in protein aggregation in living cells. For example, rapid overexpression of recombinant proteins in bacteria can lead to the formation of inclusion bodies (IBs), and this phenomenon has attracted considerable interest in recent decades. Targeting recombinant proteins into IBs provides an interesting approach for their production because they are proposed as an almost pure source of recombinant proteins. Moreover, expression in IBs provides an alternative strategy for protecting recombinant proteins from proteolytic degradation, leading to increased expression levels (1).

Although formation of IBs is common in heterologous protein expression systems, especially under overexpression conditions, a robust fusion partner is often necessary. Newly expressed proteins may exist in three states: properly folded, partially folded intermediates, and aggregated *in vivo*, which are in dynamic equilibrium and subjected to the action of chaperones and proteases (2). Recombinant proteins that exist primarily as partially folded or misfolded intermediates can be rapidly degraded *in vivo*. Thus, their accumulation can be improved significantly by shifting the equilibrium toward the formation of IBs to favor protection from proteolytic degradation. For a given protein, it is often difficult to assess which insoluble fusion partner is the most effective at targeting it into IBs. Nevertheless, some insoluble fusion partners have been demonstrated to work well with multiple target peptides or proteins, such as ketosteroid isomerase (KSI), EDDIE, and PagP (1).

When overexpressed in Escherichia coli, fungal prion HET-s accumulated predominantly in IBs with high β-sheet content and displayed very similar characteristics to amyloid proteins. The kinetics of HET-s amyloid fibril formation demonstrated that amyloid growth was nucleation dependent (3). Accumulating evidence has shown that IBs are highly ordered aggregates that bear a characteristic cross β structure similar to amyloid fibers. As occurs for amyloids, formation of IBs is promoted by intermolecular interactions in a nucleation-dependent manner through hydrophobic protein patches (4). Mutation of Pro-102 or Pro-105 to leucine in human prion (PrP) can greatly promote the formation of PrP aggregates since proline residues normally disfavor the formation of β-sheet conformation (5). IBs have been recognized as a valuable model to understand protein aggregation in eukaryotes and search for specific inhibitors or disaggregation approaches (6).

As important effectors of the innate immune system in multicellular organisms, antimicrobial peptides (AMPs) protect their hosts against a large variety of invading pathogens. Besides their antimicrobial activities, some AMPs are also recognized for their immunomodulatory properties (7). Recombinant expression of AMPs in E. coli faces two challenges; AMPs, rich in basic amino acid residues, are highly susceptible to proteolytic degradation due to their unordered structure, and they can be toxic to the producing hosts. To overcome both obstacles, a commonly used strategy is to attach them to fusion tags (8, 9). Targeting AMPs to IBs is believed to be more effective than their soluble fusion expression for masking their toxic effects and protecting them from proteolytic degradation (1, 10). For a given AMP, selecting a suitable fusion tag to confer efficient expression in IBs remains empirical or needs to be screened. Additionally, the repertoire of IB-targeted fusion tags also needs to be expanded.

Development of effective methods for recombinant production and purification of AMPs is of practical significance (11). In the present work, aggregation “hot spots” (HSs) of the fusion tag PagP were analyzed. Based on structural analysis, we demonstrated that the C-terminal region of PagP contains numerous HSs and could function as an effective fusion tag to target AMPs to IBs. Furthermore, our data also showed that mutation of proline residues in or near the aggregation HSs to hydrophobic residues could significantly improve its potency as an IB-targeting fusion tag. Fewer examples have been presented to demonstrate the separation of recombinant target proteins expressed in IBs fused with a tag. Herein, we describe an alternative approach to recover authentic AMPs expressed as IBs in fusion with PagP or its refined version.

## RESULTS

### Targeting recombinant AMPs to IBs using the PagP fusion tag.

**FIG 1:**
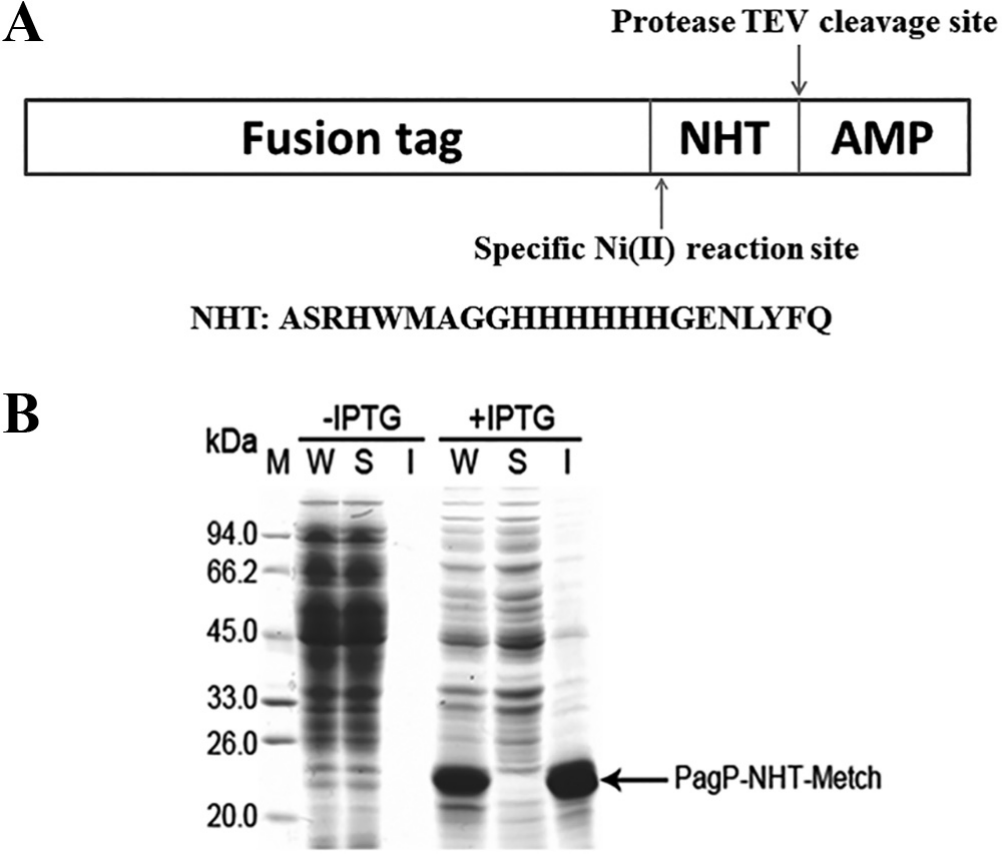
Schematic representation of the artificial peptide NHT (A) and SDS-PAGE analysis of the expression of recombinant fusion protein PagP-NHT-Metch (B). W, whole-cell lysate; S, soluble supernatant after sonication; I, inclusion bodies.

Unfavorable patterns of codon usage can affect high-level expression of recombinant proteins (12). The Metch, Andropin, and Mag II AMP genes were designed with a codon pattern adapted to the codon usage bias of E. coli using the web platform at http://genomes.urv.es/OPTIMIZER (13). Meanwhile, an artificial short peptide (NHT) with three functional motifs (motifs I to III) was designed and appended at the N terminus of AMPs (Fig. 1A). Motif I (ASRHWMAG) allows the fusion proteins to be hydrolyzed site-specifically by Ni^2+^ ions; motif II (HHHHHH) allows the fusion AMPs (NHT-Metch, NHT-Andropin, and NHT-Mag II) to be purified by Ni-chelating chromatography; and motif III (ENLYFQ) allows the fusion AMPs to be cleaved site-specifically by tobacco etch virus (TEV) protease to release authentic Metch, Andropin and Mag II.

**FIG 2:**
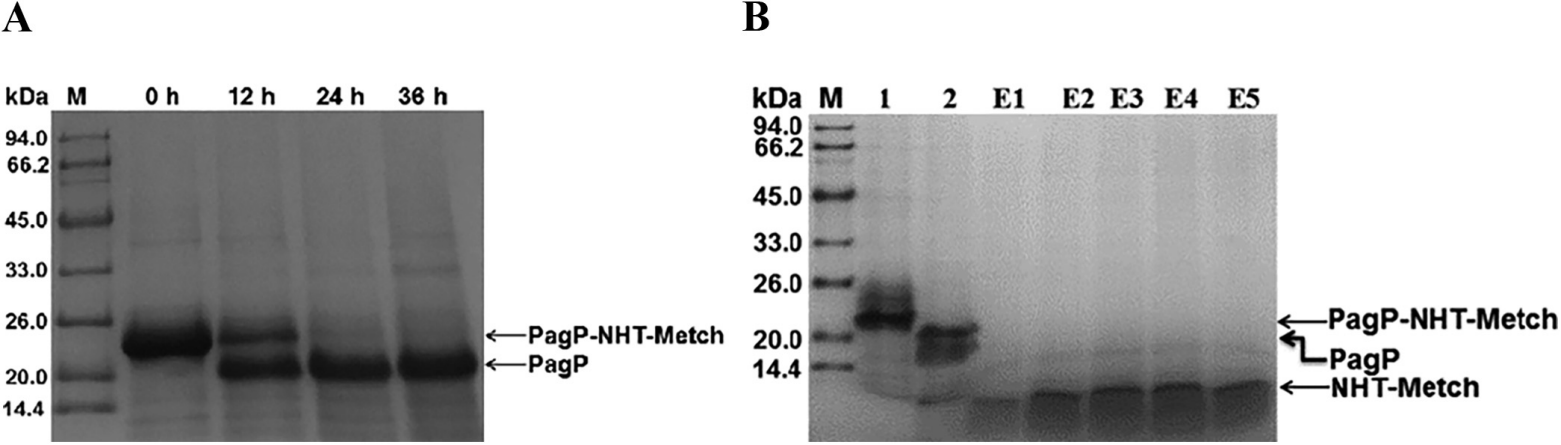
Ni2+-catalyzed cleavage of the fusion protein PagP-NHT-Metch and purification of the fusion peptide NHT-Metch. (A) Tris-SDS-PAGE analysis. Under denaturing conditions, the fusion protein PagP-NHT-Metch was subjected to Ni^2+^-catalyzed cleavage for 12, 24, and 36 h at 60°C. (B) Tricine-SDS-PAGE analysis. Lane 1, fusion protein PagP-NHT-Metch; lane 2, products after Ni^2+^-catalyzed cleavage; lanes E1 to E5, fusion peptide NHT-Metch purified by Ni-chelating affinity chromatography.

The PagP accumulates in IBs when overexpressed in E. coli (1). As expected, the PagP-NHT-Metch fusion protein accumulated predominantly in IBs (Fig. 1). Insoluble fusions in IBs are refractory to site-specific cleavage by proteases such as thrombin and TEV. An optimized amino acid sequence has been developed for Ni (II)-catalyzed cleavage (14), and it has been demonstrated that nearly complete nickel-catalyzed hydrolysis of fusion proteins can be achieved under denaturing conditions (15). Full cleavage of the PagP-NHT-Metch fusion protein was achieved after 24 h of hydrolysis at 60°C, confirmed by the appearance of the PagP fusion tag (Fig. 2A). Consequently, the released soluble His6-tagged passenger peptide NHT-Metch could be purified by Ni-affinity chromatography (Fig. 2B).

### Refinement of the PagP Tag to target recombinant peptides more efficiently to IBs.

**FIG 3:**
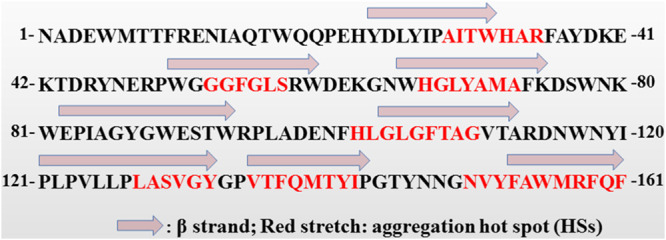
Schematic representation of the β-strands in PagP reported previously (25), and the aggregation hot spots (HSs) predicted by web-based software AGGRESCAN.

**FIG 4:**
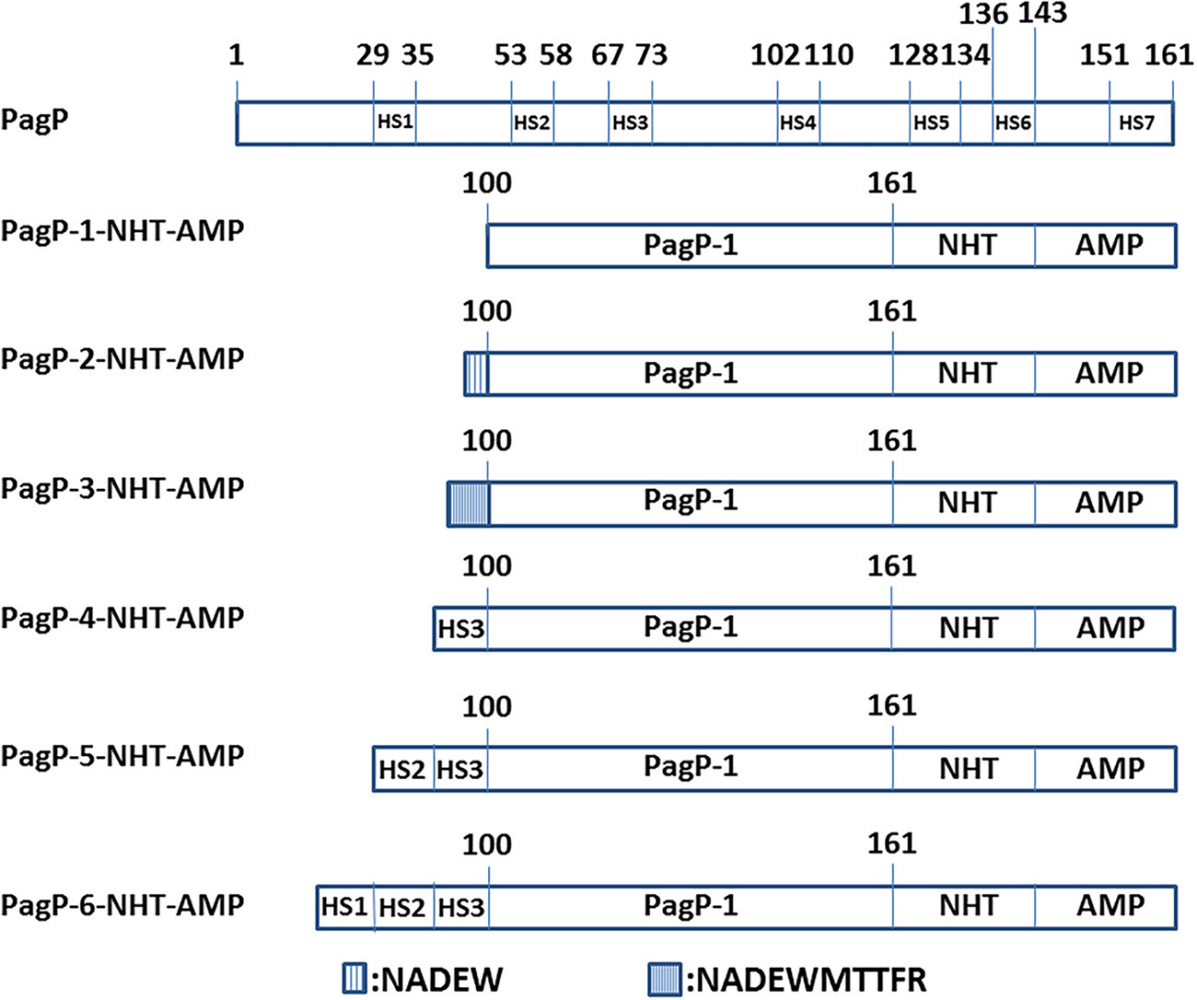
Schematic representation of the construction of fusion proteins.

**FIG 5:**
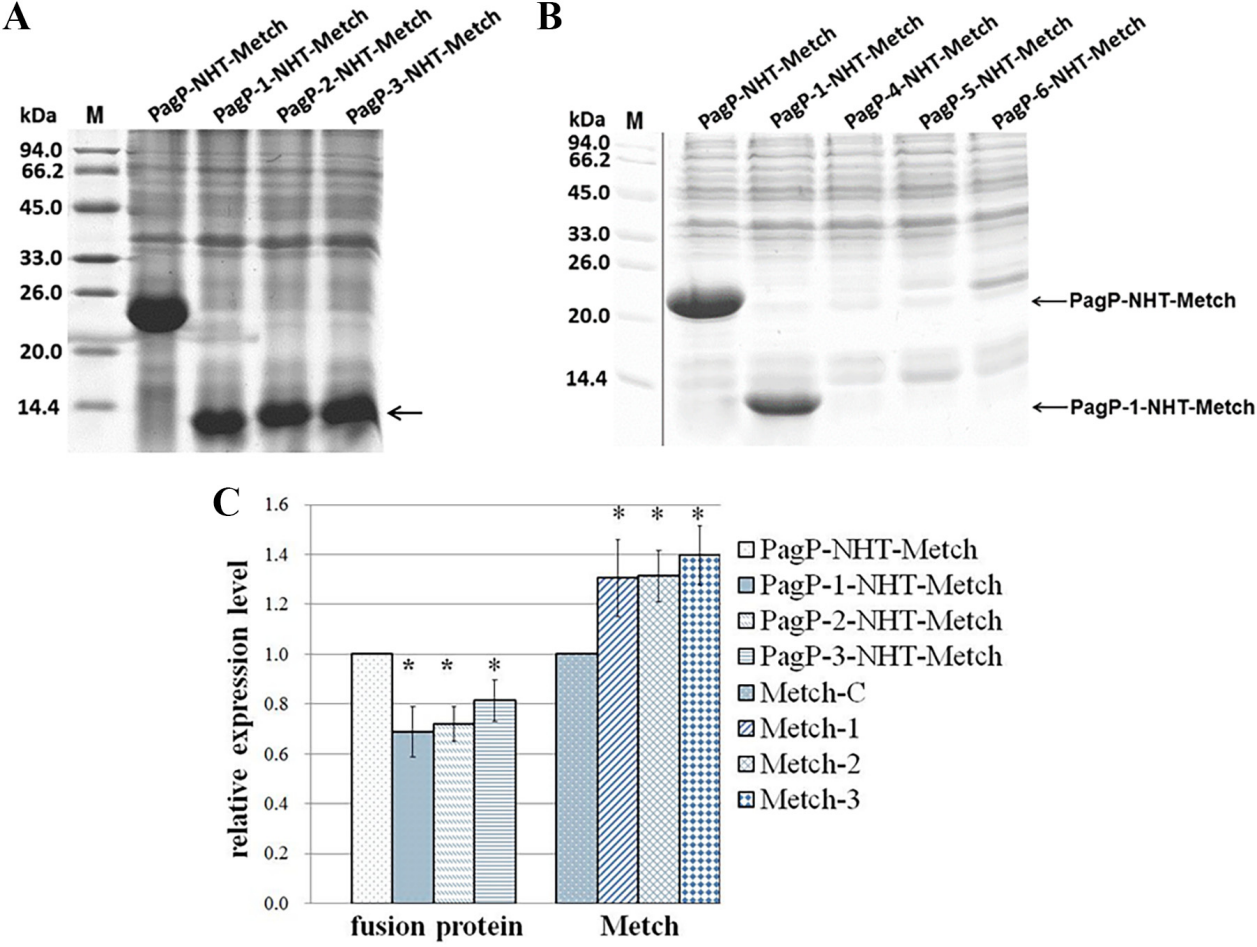
Antimicrobial peptide Metch targeted to IBs upon fusion with different versions of PagP. (A and B) Expression of six fusion proteins was analyzed by SDS-PAGE. (C) The densitometric value of the PagP-NHT-Metch band was set as 1. Each bar represents mean ± standard error from three replicates (*, *P* < 0.05). The yields of recombinant Metch from fusion proteins were calculated according to their corresponding molecular weight; for Metch-C, the yield of recombinant Metch expressed as the PagP-NHT-Metch fusion protein was as 1; Metch-1, the yield of recombinant Metch expressed as the PagP-1-NHT-Metch fusion protein; Metch-2, the yield of recombinant Metch expressed as the PagP-2-NHT-Metch fusion protein; Metch-3, the yield of recombinant Metch expressed as the PagP-3-NHT-Metch fusion protein. The molecular weight of PagP, PagP-1, PagP-2, and PagP-3 is 18.98, 6.87, 7.50, and 8.14 kDa, respectively.

As an integral outer membrane protein, E. coli PagP exhibits a β-barrel architecture with a hydrophobic exterior facing the membrane bilayer and a hydrophilic interior tunnel (16). Many of these HSs have been characterized in proteins governing neurodegenerative and systemic amyloidogenic diseases (17). Based on the aggregation-propensity tendencies for natural amino acids derived from *in vivo* experiments, the web-based AGGRESCAN software has been developed for prediction of aggregation-prone segments in protein sequences (18). To identify the putative aggregation HSs of the PagP fusion tag, we analyzed its sequence with AGGRESCAN web tools, and seven HSs (HS1 to HS7) were identified (Fig. 3). We also found that these HSs were distributed unevenly along the amino acid sequence, with four HSs present in the C-terminal region comprising residues 101 to 161. This finding implied that the C-terminal region of PagP has great potential to aggregate. We reconstructed a series of fusion tags using the C-terminal region of PagP as the template. Peptide NHT-Metch was then separately fused with these constructed tags (Fig. 4). When targeted for expression in E. coli, PagP-1-NHT-Metch, PagP-2-NHT-Metch, and PagP-3-NHT-Metch accumulated in considerable amounts (Fig. 5A). However, there was no obvious accumulation of fusion proteins for tags PagP-4, PagP-5, and PagP-6 (Fig. 5B), which possessed a denser distribution of aggregation HSs owing to addition of HS3 or a combination of HS3 with HS2 and HS1. Our results showed that a simple combination or addition of aggregation HSs to a given fusion tag might bring about the negative effect considering its ability to target passenger peptides to IBs. Compared with PagP-NHT-Metch, accumulation of PagP-1-NHT-Metch decreased in terms of overall quantity. However, the absolute yield of the recombinant peptide increased by ~40% due to the decreased molecular weight of the fusion tag PagP-3 (Fig. 5C).

**FIG 6:**
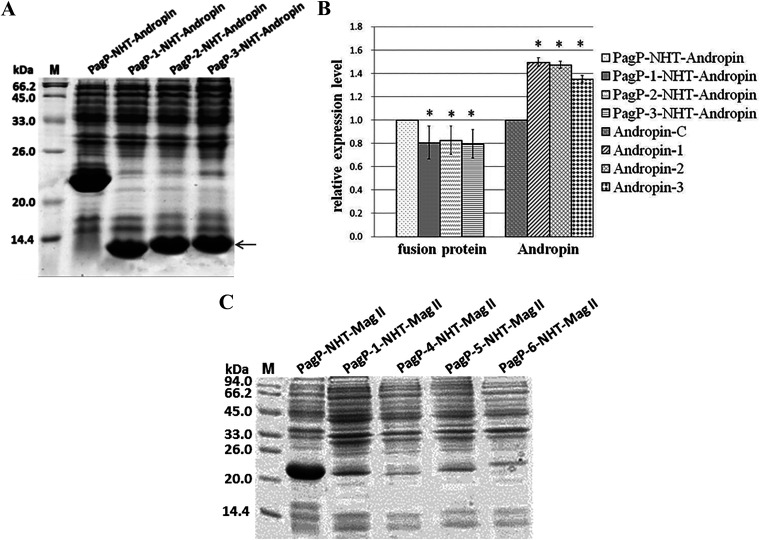
Expression of antimicrobial peptides Mag II and Andropin in fusion with refined versions of PagP. (A) Andropin was fused to PagP-1, PagP-2, and PagP-3, and expression was analyzed by SDS-PAGE. (B) The densitometric value of the PagP-NHT-Andropin band was set as 1. Bars represent mean ± standard error from three replicates (*, *P* < 0.05). The yield of recombinant Andropin from fusion proteins was calculated according to their corresponding molecular weight. Andropin-C, the yield of recombinant Andropin expressed as the PagP-NHT-Andropin fusion protein was set as 1; Andropin-1, the yield of recombinant Andropin expressed as the PagP-1-NHT-Andropin fusion protein; Andropin-2, the yield of recombinant Andropin expressed as the PagP-2-NHT-Andropin fusion protein; Andropin-3, the yield of recombinant Andropin expressed as the PagP-3-NHT-Andropin fusion protein. (C) Mag II was fused to PagP, PagP-1, PagP-4, PagP-5, and PagP-6, and expression was analyzed by SDS-PAGE.

We then fused NHT-Andropin and NHT-Mag II with these tags. All fusion tags targeted NHT-Andropin to IBs effectively, and likewise for NHT-Metch, increasing the absolute yield of recombinant Andropin up to almost 50% (Fig. 6B and B). However, these fusion tags lost their efficacy when fused with passenger NHT-Mag II, the accumulation of which was barely detected by SDS-PAGE (Fig. 6C). Our results suggest that the effectiveness of a given fusion tag for IB-targeted expression was significantly affected by the physicochemical properties of the passenger proteins.

### Mutation of PagP for more efficient formation of IBs.

**FIG 7:**
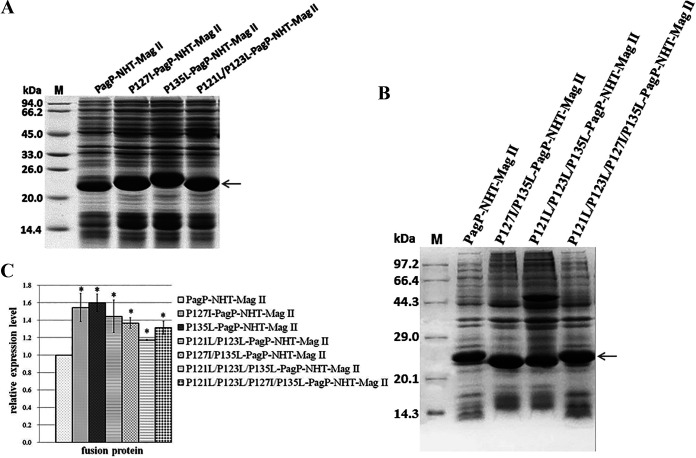
Mutation of the PagP tag for efficient formation of IBs. (A and B) NHT-Mag II was fused to the different mutant PagPs, and expression was analyzed by SDS-PAGE. (C) The densitometric value of the PagP-NHT-Mag II band was set as 1. Bars represent mean ± standard error from three replicates (*, *P* < 0.05).

Recombinant IBs in E. coli possess the characteristic cross-β structure of amyloid fibers (19). Changing the hydrophobicity or propensity to form a β-strand may affect the aggregation of a given protein when overexpressed. We analyzed the secondary structure of PagP and highlighted its C-terminal region, comprising four β-sheet elements (Fig. 3) (16). We found that this region is rich in proline residues (P-121, P-123, P-127, and P-135), which are scarce in β-sheet structure. Compared with proline, isoleucine or leucine has a higher propensity to form the β-sheet (20). Moreover, isoleucine and leucine have bulky hydrophobic side chains that favor intermolecular and intramolecular interactions between side chains, facilitating the formation of aggregates. We replaced Pro with Leu or Ile to favor the formation of β-strands. A series of mutants (P127I-PagP-NHT-Mag II, P135L-PagP-NHT-Mag II, P121L/P123L-PagP-NHT-Mag II, P127I/P135L-PagP-NHT-Mag II, P121L/P123L/P135L-PagP-NHT-Mag II, and P121L/P123L/P127I/P135L-PagP-NHT-Mag II) were constructed. When overexpressed in E. coli, these mutants accumulated in greater quantities compared with PagP-NHT-Mag II. The yields of these recombinant mutants were increased by up to 44.3 to 60.5% (Fig. 7). These results demonstrated that the aggregation ability of a fusion tag could be further improved by increasing its overall hydrophobicity or enhancing its propensity to form a more stable β-sheet conformation. Nevertheless, the ability of PagP to aggregate was not further improved by increasing its overall hydrophobicity or propensity to form stable β-strands. The accumulation level of group I (P127I/P135L-PagP-NHT-Mag II, P121L/P123L/P135L-PagP-NHT-Mag II, and P121L/P123L/P127I/P135L-PagP-NHT-Mag II mutants) decreased slightly in comparison with that of group II (P127I-PagP-NHT-Mag II, P135L-PagP-NHT-Mag II, or P121L/P123L-PagP-NHT-Mag II mutants; Fig. 7).

**FIG 8:**
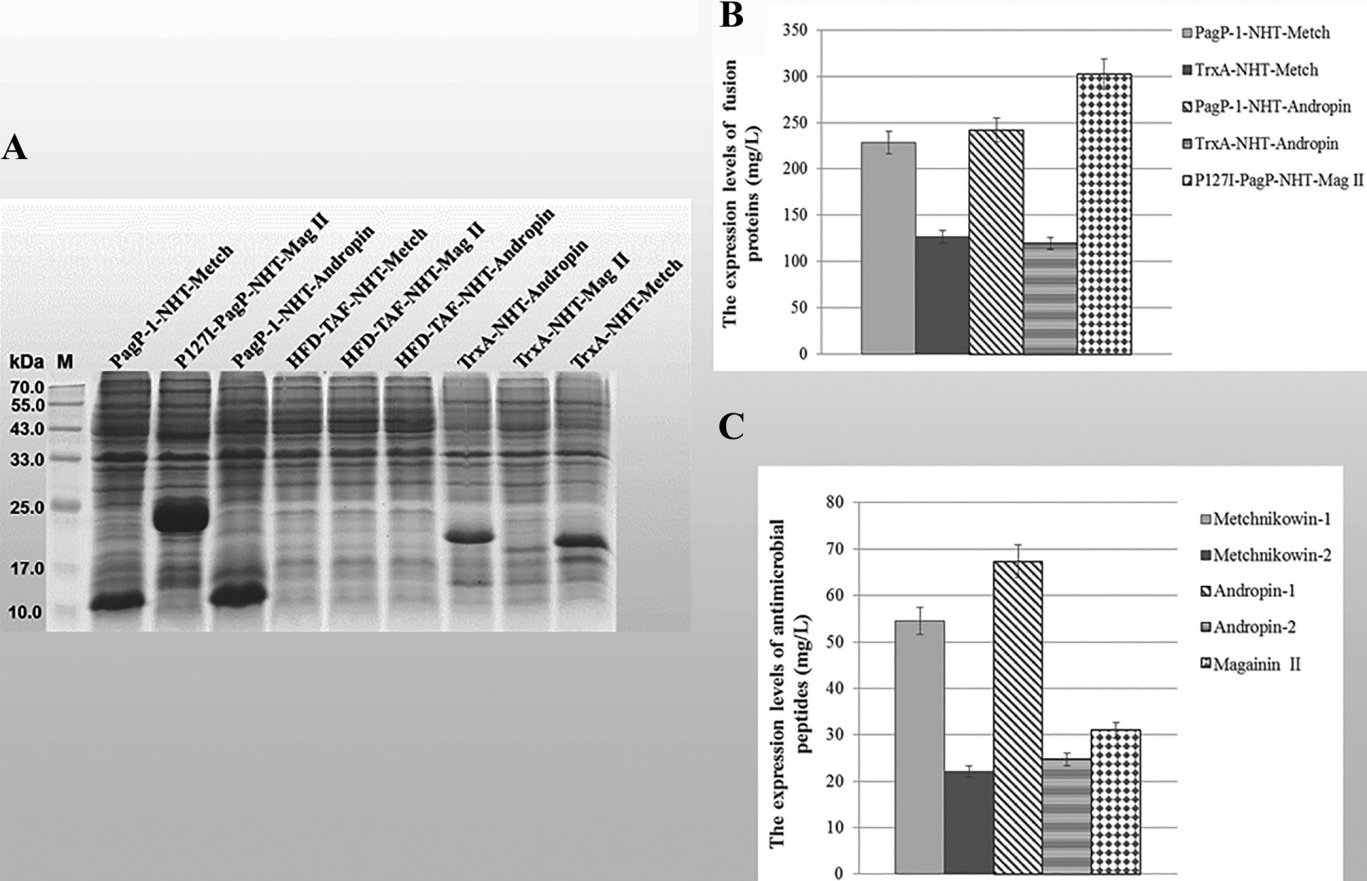
Expression of antimicrobial peptides Mag II, Metch and Andropin in fusion with PagP-1, P127I-PagP, TrxA and HFD-TAF, respectively. (A) The expression of fusion proteins was analyzed by SDS-PAGE; (B) expression levels of fusion proteins; (C) expression levels of antimicrobial peptides. Metchnikowin-1: the expression level of Metchnikowin in fusion with PagP-1; Metchnikowin-2: the expression level of Metchnikowin in fusion with TrxA; Andropin-1: the expression level of Andropin in fusion with PagP-1; Andropin-2: the expression level of Andropin in fusion with TrxA.

We also constructed another series of fusion proteins for recombinant expression of AMPs, using solubility-enhancing tag Thioredoxin A (TrxA) and insolubility-targeting tag the histone fold domain (HFD) of the human transcription factor TAF12 (HFD-TAF) (21, 22), respectively. As shown in Fig. 8A, the fusion protein TrxA-NHT-Metch and TrxA-NHT-Andropin could accumulate in considerable quantities, while TrxA-NHT-Mag II could not. Moreover, the tag HFD-TAF could not target the three antimicrobial peptides (Metch, Andropin, Mag II) to accumulate in insoluble aggregates. Compared with refined version of PagP (PagP-1, P127I-PagP), the tag TrxA and HFD-TAF target the AMPs (Metch, Andropin, Mag II) to recombinantly express much less efficiently (Fig. 8B). When being fused with PagP-1 or P127I-PagP, the expression levels of Metch, Andropin, and Mag II reach up to 54.53 mg/L, 67.37 mg/L, and 31.01 mg/L, respectively (Fig. 8C).

### Purification of recombinant AMPs and analysis of biological activities.

**FIG 9:**
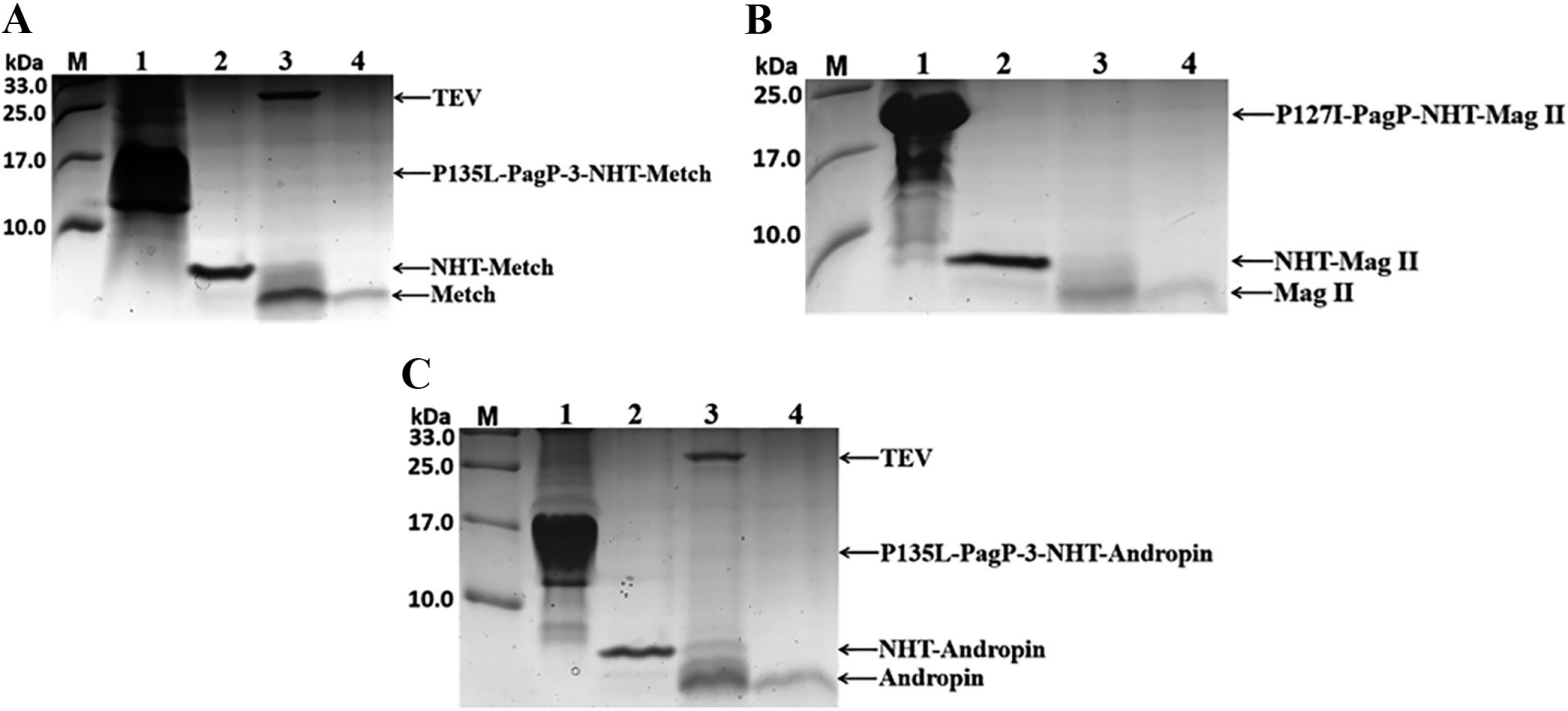
Purification of recombinant antimicrobial peptides. (A) Lane 1, fusion protein PagP-3-NHT-Metch; lane 2, purified NHT-Metch; lane 3, products of NHT-Metch targeted for site-specific cleavage by TEV protease; lane 4, purified antimicrobial peptide Metch. (B) Lane 1, fusion protein P135L-PagP-NHT-Mag II; lane 2, purified NHT-Mag II; lane 3, products of NHT-Mag II targeted for site-specific cleavage by TEV protease; lane 4, purified antimicrobial peptide Mag II. (C) Lane 1, fusion protein PagP-3-NHT-Andropin; lane 2, purified NHT-Andropin; lane 3, products of NHT-Andropin targeted for the site-specific cleavage by TEV protease; lane 4, purified antimicrobial peptide Andropin.

**FIG 10:**
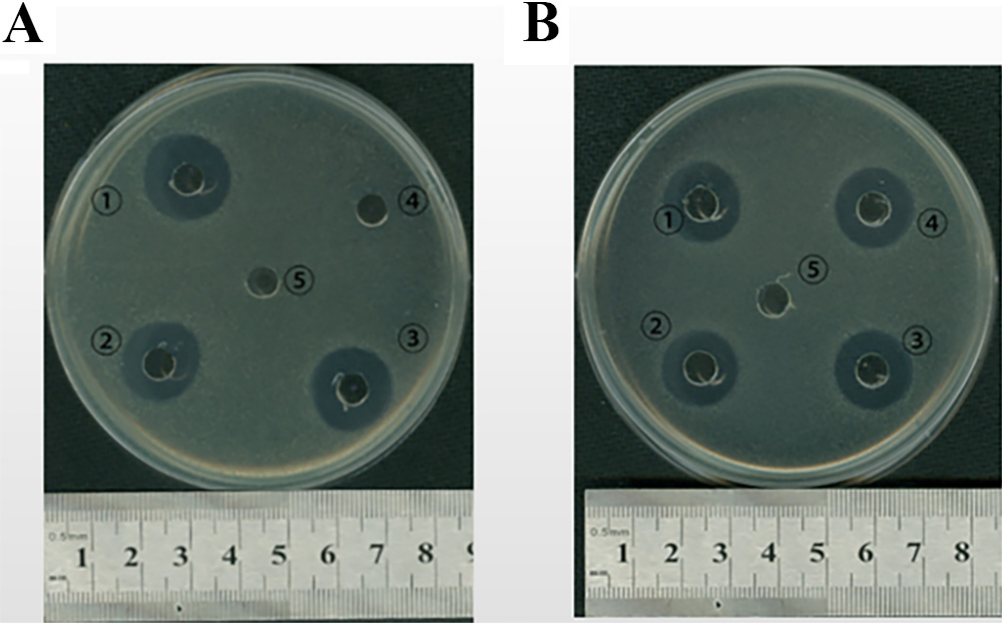
Antibacterial activity assay of recombinant antimicrobial peptides against Gram-positive S. aureus ATCC 25923 (A) and Gram-negative E. coli MG1655 (B) based on appearance of inhibition zones. (1) 5.00 μg of commercial chemosynthetic antimicrobial peptide Magainin II; (2) 2.65 μg of purified recombinant antimicrobial peptide Magainin II; (3) 4.58 μg of purified recombinant antimicrobial peptide Andropin. (4) 5.54 μg of purified recombinant antimicrobial peptide Metchnikowin; (5) 25 μL of phosphate buffer solution (50 mM, pH 7.5).

Fusion tags must normally be cleaved and removed due to their potential to interfere with the activities of passenger proteins. This is especially challenging in the case of IB-targeted expression since IBs are usually solubilized in harsh denaturants or detergents, precluding the utilization of enzymatic cleavage (for example, with TEV protease or thrombin). With the help of the artificially designed peptide NHT, recombinant AMPs in fusion with NHT could be conveniently purified by Ni-affinity chromatography after Ni^2+^-catalyzed specific cleavage. Eventually, authentic AMPs were recovered following successive site-specific cleavage by TEV protease and ion-exchange chromatography (Fig. 9). Mag II and Andropin exhibited biological activity against both Gram-positive and Gram-negative bacteria (23, 24), while Metch showed inhibitory activity toward Gram-positive bacteria (25). We then tested antimicrobial activity using classical inhibition zone assays. As expected, the appearance of clear inhibition zones confirmed that the recombinant peptides possessed their native antimicrobial activities against both Gram-positive S. aureus and Gram-negative E. coli, or only against Gram-positive S. aureus in the case of Metch (Fig. 10) (Table 1).

**TABLE 1:** Diameters of inhibitory zone by antimicrobial peptides^*a*^

Antimicrobial peptides	Escherichia * coli*	Staphylococcus aureus
Mag II-1	1.55 ± 0.09 cm	1.48 ± 0.08 cm
Mag II-2	1.43 ± 0.04 cm	1.30 ± 0.12 cm
Metch	-	1.28 ± 0.08 cm
Andropin	1.33 ± 0.04 cm	1.30 ± 0.07 cm
Phosphate-buffered solution (50 mM, pH 7.5)	-	-

a -: No inhibitory zone.

## DISCUSSION

Compared with solubility-enhancing tags, IB-targeting fusion partners are believed to be more effective at protecting passenger proteins from proteolytic degradation and masking their toxic activities. Using a given fusion tag as the template, there are few examples illustrating how to refine its sequence to increase the yields of passenger proteins. It has been proposed that the primary structure of a polypeptide intrinsically determines its propensity to aggregate (26). Moreover, some short specific amino acid stretches play crucial roles as the initial nucleators (HSs) during aggregation. The finding of unevenly distributed HSs at the C-terminal region of PagP prompted us to investigate whether this C-terminal region alone could retain high potency to direct passenger peptides to IBs. In fact, the increased absolute yields of passenger peptides implied that a larger number of molecules formed aggregates with truncated PagP, reinforcing its potency to form aggregates.

A larger fusion tag implies a lower peptide-to-tag ratio, disfavoring the final yield of target peptides (8). Screening and identification of effective shorter fusion tags is therefore of practical significance. Nevertheless, we cannot ignore the fact that these versions of PagP lost their ability to efficiently target Mag II to IBs. Some IBs have been shown to have considerable biological activity, characterized by a loose arrangement of protein molecules (27). Mag II is a very potent AMP that exerts its biological activity by destroying the integrity of the cytoplasmic membrane (19). When fused with truncated PagP (PagP-1, PagP-2, or PagP-3), the resulting fusion proteins might retain partial antimicrobial activity or be unable to form stable aggregates immediately, making them more vulnerable to proteolytic degradation induced by multiple levels of cellular responses.

Bacterial IBs have been found to possess some amyloid-like properties and contain similar structures (4, 6). Much effort has been made to understand the molecular mechanisms underlying amyloid cascades (28). Aβ 42 is an amyloidogenic peptide in which a central hydrophobic stretch (residues 17 to 21) is predicted to be an aggregation HS (18). Decreasing hydrophobicity or β-sheet propensity in this stretch could significantly affect aggregation propensity and neurotoxicity (29, 30). Besides, the presence of proline residues is believed to decrease the overall protein aggregation propensity (31). A hydrophobic region (residues 58 to 63) was identified in Microcin E492. Compared with wild-type MccE492, mutants P57A and P59A exhibited a greater tendency to form amyloid aggregates *in vivo* and aggregated significantly faster *in vitro* (32). PagP is an outer membrane protein rich in β-strands that readily accumulates in IBs when expressed in E. coli cytoplasm (16, 33). We found that there are several proline residues (P121, P123, P127, and P135) inside or near the β8 strand (Fig. 3). Leu and Ile are more hydrophobic and have higher propensity to form β-strands than Pro (20). These findings prompted us to construct mutants P121L/P123L-PagP-NHT-Mag II, P127I-PagP-NHT-Mag II, and P135L-PagP-NHT-Mag II to further increase their insoluble accumulation. For newly synthesized recombinant proteins, soluble properly folded forms, partially folded intermediates, and insoluble aggregates coexist in a dynamic equilibrium (1). Partially folded forms are vulnerable to proteolytic degradation. These mutants might aggregate more rapidly, bringing about enhanced resistance to proteolytic degradation. This hypothesis is further evidenced by the scarce accumulation of recombinant fusion protein PagP-1-NHT-Mag II. Similar reasons can account for the unsuccessful expression of HFD-TAF fusions. The increased accumulation levels demonstrated that the aggregation capability of a given IB-targeted fusion tag could be improved significantly by properly enhanced hydrophobicity or propensity to form β-strands (Fig. 7). To our knowledge, few previous reports have demonstrated that a fusion tag can be successfully improved to form aggregates in E. coli.

Many linear AMPs have an undefined structure in aqueous solution and are highly susceptible to proteolytic degradation during heterogeneous expression (34). Targeting AMPs to IBs is preferable to protect them from cellular proteases and mask their cellular toxicity (35). However, fusion tag removal is often necessary. The soluble fusion proteins are tractable to site-specific cleavage by some proteases such as thrombin or TEV protease (36). However, this strategy may not be a feasible option when fusion proteins exist in insoluble Ibs. In this setting, fusion tag removal is biochemically challenging. Few examples have illustrated how to separate target peptides from fusion proteins expressed in Ibs. A previous report has described a method for purification of the target proteins expressed in fusion with PagP using nickel ion-catalyzed peptide bond hydrolysis (37). Even so, this method is not applicable to purification of the short peptides. In this work, we designed a NHT linker sequence inserted between the fusion tag and AMPs (Fig. 1). This artificial peptide possesses three functional motifs, allowing specific Ni (II)-catalyzed cleavage, Ni-chelating affinity chromatography, and TEV protease-mediated specific cleavage to be performed successfully. This approach is particularly applicable to production of short unstructured peptides. We believe that the approach presented herein will promote IB-targeted expression of short peptides.

## MATERIALS AND METHODS

### Bacterial strains and plasmid construction.

The E. coli DH5α strain was used as a host for gene cloning and preparation of plasmids, while E. coli BL21(DE3) was used as a host strain for expression of recombinant proteins. All E. coli strains were cultured in LB medium supplemented with antibiotics as needed.

Antimicrobial peptides magainin II (Mag II) (23), Metchnikowin (Metch) (25) and Andropin (24) were selected as test cases for this study. Mag II, Metch, and Andropin genes were synthesized by overlap extension PCR according to their amino acid sequences, with a codon pattern adapted to the usage bias of E. coli (13, 38). The Mag II gene was first synthesized by overlap extension PCR with primers 1 to 4, then sequentially amplified with primers 8 and 4, primers 9 and 4, and primers 10 and 4. The resulting amplicon encoded a Mag II fusion peptide with a sequence (ASRHWMAG) for Ni(II)-dependent peptide bond hydrolysis (39), a His_6_ tag (HHHHHH) and a recognition site (ENLYFQ) for the site-specific protease tobacco etch virus (TEV) (40) at its N terminus. The resulting sequence ASRHWMAGHHHHHHENLYFQ was denoted NHT.

The Metch gene was also first synthesized by overlap extension PCR with primers 5 to 7, then sequentially amplified with primers 8 and 7, primers 9 and 7, and primers 10 and 7. The resulting amplicon encoded an NHT-Metch fusion peptide.

Similarly, the Andropin fusion gene was first synthesized by overlap extension PCR with primers 11 to 15, then sequentially amplified with primers 8 and 15, primers 9 and primer 15, and primers 10 and 15. The Andropin fusion gene was finally constructed, encoding the NHT-Andropin fusion peptide. These fusion AMPs are outlined in Fig. S1 in the supplemental material. The PagP gene (Gene ID: 946360) encoding the mature form was amplified from the chromosome of E. coli strain MG1655 using primers 16 and 17. The primers designed for gene amplification and synthesis are listed in Table S1.

PagP is a Gram-negative bacterial outer membrane protein and extremely prone to accumulating in IBs when overexpressed in E. coli (1). Based on analysis of the PagP sequence using the web platform AGGRESCAN at http://bioinf.uab.es/aggrescan/ (18), seven aggregation HSs were identified and denoted HS1 to HS7. The C-terminal region of PagP (residues 101 to 161) was denoted PagP-1, comprising four aggregation HSs (HS4 to HS7; Fig. 3 and 4).

After being digested by EcoRI and HindIII, the Metch fusion gene was inserted into expression vectors pET30a and pET28b, generating pET30a-NHT-Metch and pET28b-NHT-Metch constructs, respectively. The amplicon encoding mature PagP was digested with NdeI and BamHI, then inserted into pET30a-NHT-Metch, generating pET30a-PagP-NHT-Metch for intracellular expression of the fusion protein PagP-NHT-Metch in E. coli. Similarly, after being digested by EcoRI and HindIII, the NHT-Mag II gene and NHT-Andropin gene were inserted into the expression vector pET30a, generating pET30a-NHT-Mag II and pET30a-NHT-Andropin, respectively. The mature PagP gene was digested with NdeI and BamHI, then inserted into pET30a-NHT-Mag II and pET30a-NHT-Andropin, generating pET30a-PagP-NHT-Mag II and pET30a-PagP-NHT-Andropin, respectively, for intracellular expression of the fusion proteins PagP-NHT-Mag II and PagP-NHT-Andropin in E. coli.

Using the pET30a-PagP-NHT-Metch construct as the template, a fragment encoding fusion protein PagP-1-NHT-Metch was amplified with primers 18 and 7 and inserted into the pET28b vector, generating pET28b-PagP-1-NHT-Metch. Similarly, corresponding fragments were amplified using primers 19 and 7, primers 20 and 7, primers 21 and 7, primers 22 and primer 7, primers 23 and 7, and primers 24 and 7, and separately inserted into the pET28b vector, generating pET28b-PagP-2-NHT-Metch, pET28b-PagP-3-NHT-Metch, pET28b-PagP-4-NHT-Metch, pET28b-PagP-5-NHT-Metch, and pET28b-PagP-6-NHT-Metch, respectively. Furthermore, the fragment encoding NHT-Mag II was substituted for the NHT-Metch gene, generating the corresponding expression vectors pET28b-PagP-1-NHT-Mag II, pET28b-PagP-2-NHT-Mag II, and pET28b-PagP-3-NHT-Mag II. Similarly, the fragment encoding NHT-Andropin was substituted for the NHT-Metch gene, generating the corresponding expression vectors pET28b-PagP-1-NHT-Andropin, pET28b-PagP-2-NHT-Andropin, and pET28b-PagP-3-NHT-Andropin.

The Thioredoxin A (TrxA) (Gene ID: 948289) gene was amplified from the chromosome of E. coli strain MG1655 using primers 25 and 26, and digested with NdeI and BamHI, then inserted into pET30a-NHT-Metch, pET30a-NHT-Mag II and pET30a-NHT-Andropin, generating pET30a-TrxA-NHT-Metch pET30a-TrxA-NHT-Mag II and pET30a-TrxA-NHT-Andropin, respectively, for intracellular expression of the fusion proteins TrxA-NHT-Metch, TrxA-NHT-Mag II and TrxA-NHT-Andropin in E. coli. The histone fold domain (HFD) of human transcription factor TAF12 (HFD-TAF) gene was synthesized with a codon pattern adapted to the usage bias of E. coli (13, 22) (Table S1). The fragment encoding HFD-TAF was substituted for the TrxA gene, generating the corresponding expression vectors pET30a-HFD-TAF-NHT-Metch, pET30a-HFD-TAF-NHT-Mag II, and pET30a-HFD-TAF-NHT-Andropin.

All constructs were checked by DNA sequencing, and primer sequences are listed in Supplementary data Table S1.

### Site-specific mutation of PagP.

PagP mutants were generated using pET30a-PagP-NHT-Mag II as the template according to our previous report (41). The resulting constructs were denoted pET30a-P121L/P123L-PagP-NHT-Mag II, pET30a-P127I-PagP-NHT-Mag II, pET30a-P135L-PagP-NHT-Mag II, pET30a-P127I/P135L-PagP-NHT-Mag II, pET30a-P121L/P123L/P135L-PagP-NHT-Mag II, and pET30a-P121L/P123L/P127I/P135L-PagP-NHT-Mag II. In the case of P121L/P123L-PagP, Pro at position 121 and 123 was mutated to Leu using primers 27 and primer 28. Similarly, the P127I-PagP mutant was constructed using primers 29 and 30, and the P135L-PagP mutant was constructed using primers 31 and 32. These mutants were also used as the templates for further mutation. Following the second and third rounds of mutation, P127I/P135L-PagP, P121L/P123L/P135L-PagP and P121L/P123L/P127I/P135L-PagP mutants were constructed. All mutations were checked by DNA sequencing.

### Expression of fusion proteins.

E. coli BL21(DE3) cells harboring expression vectors were cultured overnight at 37°C in 6 mL of LB medium. Cultures were then diluted 100-fold and cultured at 37°C until they reached mid-log phase (optical density at 600 nm [OD_600_] ~0.6 to 0.8). Expression of fusion proteins was induced by adding isopropyl β-D-1-thiogalactopy-ranoside (IPTG) to a final concentration of 0.3 mM, and cultures were further incubated for 12 h at 37°C. A 100-mL sample of culture was centrifuged for 10 min at 6,000 rpm and 4°C to harvest bacteria. The E. coli cells were resuspended in 10 mL lysis buffer (50 mM NaH_2_PO_4_-Na_2_HPO_4_, 0.2 M NaCl, 20 mM imidazole, pH 8.0), then lysed by sonication on ice. IBs were isolated by centrifugation for 15 min at 12,000 rpm at 4°C, washed twice with washing buffer I (20 mM Tris-HCl, 50 mM NaCl, 0.1% Triton X-100, 5 mM EDTA, pH 8.0), then with washing buffer II (20 mM Tris-HCl, 50 mM NaCl, pH 8.0). Extracted IBs and whole-cell lysates were subjected to analysis by SDS-PAGE, and gel images were further analyzed using Image Lab software (BIO-RAD) to evaluate expression levels of fusion proteins.

### Specific hydrolysis of fusion proteins by Ni (II) ions.

Specific hydrolysis of fusion proteins by Ni (II) ions was conducted under denaturing conditions according to a previous report (15). Isolated IBs were dissolved in hydrolysis reaction buffer (20 mM HEPES, 6 M GuHCl, pH 8.2) at a concentration of ~200 μM, and fusion proteins were subjected to hydrolysis by addition of NiSO_4_ to a final concentration of 5 mM, and incubated for 12, 24, or 36 h at 60°C, to investigate the hydrolysis process. The mixture was diluted 5-fold with addition of lysis buffer (50 mM NaH_2_PO_4_-Na_2_HPO_4_, 0.3 M NaCl, 20 mM imidazole, pH 8.0), and centrifuged for 15 min at 12,000 rpm and 4°C to remove the precipitated fusion tag.

### Purification of recombinant AMPs.

After being specifically hydrolyzed by Ni^2+^ ions, fusion proteins were split into a tag and the NHT-AMP peptide, which was further purified by Ni-chelating affinity chromatography according to the protocol specified by the manufacturer (GE Healthcare Bio-Sciences). First, the column was equilibrated with lysis buffer (50 mM NaH_2_PO_4_-Na_2_HPO_4_, 0.3 M NaCl, 20 mM imidazole, pH 8.0), and the protein sample was loaded. The loaded column was washed three times with washing buffer III (50 mM NaH_2_PO_4_-Na_2_HPO_4_, 0.3 M NaCl, 40 mM imidazole, pH 8.0), and the recombinant NHT fusion (NHT-Metch, NHT-Mag or NHT-Andropin) was eluted with elution buffer I (50 mM Na_2_HPO_4_-NaH_2_PO_4_, 250 mM imidazole, pH 7.0).

The fusion AMP (NHT-Metch, NHT-Mag or NHT-Andropin) in elution buffer I was directly subjected to specific cleavage by addition of TEV protease for 12 h at 25°C according to a previous report (40). The released AMP (Metch, Mag II or Andropin) was further purified using ion-exchange chromatography using Macro-Prep CM Resin (Bio-Rad). The column was first equilibrated with two column volumes of equilibration buffer (200 mM imidazole, 50 mM NaH_2_PO_4_-Na_2_HPO_4_, pH 7.0). A 5-mL sample of protein was loaded onto the column, and recombinant AMPs were eluted with elution buffer II (500 mM NaCl, 50 mM Na_2_HPO_4_-NaH_2_PO_4_, pH 8.0).

### Microbicidal activity assay of recombinant AMPs.

The antimicrobial activities of recombinant AMPs were analyzed by inhibition zone assay according to the protocol in a previous report (42). Briefly, Gram-positive Staphylococcus aureus ATCC 25923 and Gram-negative E. coli strain K_12_D_31_ were grown overnight at 37°C in LB medium. A 50-μL sample of culture was inoculated into 50 mL of fresh LB medium and incubated for an additional 2 to 3 h at 37°C to OD_600_ ~0.5. A 200-μL sample of cell suspension was inoculated into 50 mL of prewarmed (45°C) LB medium containing 0.8% (wt/vol) agar and rapidly dispersed. The medium was then poured into a petri dish (9 cm diameter) to form a uniform layer to a depth of ~1.5 mm. Holes with a diameter of 2 mm were punched into the gelated medium. For microbicidal activity assay, recombinant AMPs were added into the punched holes, and the plate was incubated for 12 h at 37°C to assess the appearance of inhibition zones.

### Data availability.

The original contributions presented in this study are included in the article/Supplemental Material. Further inquiries can be directed to the corresponding author.

## References

[1] Hwang PM, Pan JS, Sykes BD. 2014. Targeted expression, purification, and cleavage of fusion proteins from inclusion bodies in *Escherichia coli*. FEBS Lett 588:247–252. doi:10.1016/j.febslet.2013.09.028. 24076468

[2] Villaverde A, Carrio MM. 2003. Protein aggregation in recombinant bacteria: biological role of inclusion bodies. Biotechnol Lett 25:1385–1395. doi:10.1023/a:1025024104862. 14514038

[3] Sabate R, Espargaro A, Saupe SJ, Ventura S. 2009. Characterization of the amyloid bacterial inclusion bodies of the HET-s fungal prion. Microb Cell Fact 8:56. doi:10.1186/1475-2859-8-56. 19863787 PMC2774669

[4] Garcia-Fruitos E, Sabate R, de Groot NS, Villaverde A, Ventura S. 2011. Biological role of bacterial inclusion bodies: a model for amyloid aggregation. FEBS J 278:2419–2427. doi:10.1111/j.1742-4658.2011.08165.x. 21569209

[5] Kraus A, Anson KJ, Raymond LD, Martens C, Groveman BR, Dorward DW, Caughey B. 2015. Prion protein prolines 102 and 105 and the surrounding lysine cluster impede amyloid formation. J Biol Chem 290:21510–21522. doi:10.1074/jbc.M115.665844. 26175152 PMC4571877

[6] Ramon A, Senorale-Pose M, Marin M. 2014. Inclusion bodies: not that bad. Front Microbiol 5:56. doi:10.3389/fmicb.2014.00056. 24592259 PMC3924032

[7] Scott MG, Davidson DJ, Gold MR, Bowdish D, Hancock RE. 2002. The human antimicrobial peptide LL-37 is a multifunctional modulator of innate immune responses. J Immunol 169:3883–3891. doi:10.4049/jimmunol.169.7.3883. 12244186

[8] Li Y. 2009. Carrier proteins for fusion expression of antimicrobial peptides in *Escherichia coli*. Biotechnol Appl Biochem 54:1–9. doi:10.1042/BA20090087. 19575694 PMC7188355

[9] Chen YQ, Zhang SQ, Li BC, Qiu W, Jiao B, Zhang J, Diao ZY. 2008. Expression of a cytotoxic cationic antibacterial peptide in *Escherichia coli* using two fusion partners. Protein Expr Purif 57:303–311. doi:10.1016/j.pep.2007.09.012. 17977015

[10] Pane K, Durante L, Pizzo E, Varcamonti M, Zanfardino A, Sgambati V, Di Maro A, Carpentieri A, Izzo V, Di Donato A, Cafaro V, Notomista E. 2016. Rational design of a carrier protein for the production of recombinant toxic peptides in *Escherichia coli*. PLoS One 11:e0146552. doi:10.1371/journal.pone.0146552. 26808536 PMC4726619

[11] Wibowo D, Zhao CX. 2019. Recent achievements and perspectives for large-scale recombinant production of antimicrobial peptides. Appl Microbiol Biotechnol 103:659–671. doi:10.1007/s00253-018-9524-1. 30470869

[12] Kane JF. 1995. Effects of rare codon clusters on high-level expression of heterologous proteins in *Escherichia coli*. Curr Opin Biotechnol 6:494–500. doi:10.1016/0958-1669(95)80082-4. 7579660

[13] Puigbo P, Guzman E, Romeu A, Garcia-Vallve S. 2007. OPTIMIZER: a web server for optimizing the codon usage of DNA sequences. Nucleic Acids Res 35:W126–131. doi:10.1093/nar/gkm219. 17439967 PMC1933141

[14] Krezel A, Kopera E, Protas AM, Poznański J, Wysłouch-Cieszyńska A, Bal W. 2010. Sequence-specific Ni (II)-dependent peptide bond hydrolysis for protein engineering. Combinatorial library determination of optimal sequences. J Am Chem Soc 132:3355–3366. doi:10.1021/ja907567r. 20166730

[15] Kopera E, Belczyk-Ciesielska A, Bal W. 2012. Application of Ni (II)-assisted peptide bond hydrolysis to non-enzymatic affinity tag removal. PLoS One 7:e36350. doi:10.1371/journal.pone.0036350. 22574150 PMC3344860

[16] Hwang PM, Choy WY, Lo EI, Chen L, Forman-Kay JD, Raetz CR, Prive GG, Bishop RE, Kay LE. 2002. Solution structure and dynamics of the outer membrane enzyme PagP by NMR. Proc Natl Acad Sci USA 99:13560–13565. doi:10.1073/pnas.212344499. 12357033 PMC129713

[17] Chiti F, Dobson CM. 2006. Protein misfolding, functional amyloid, and human disease. Annu Rev Biochem 75:333–366. doi:10.1146/annurev.biochem.75.101304.123901. 16756495

[18] Conchillo-Sole O, de Groot NS, Aviles FX, Vendrell J, Daura X, Ventura S. 2007. AGGRESCAN: a server for the prediction and evaluation of “hot spots” of aggregation in polypeptides. BMC Bioinformatics 8:65. doi:10.1186/1471-2105-8-65. 17324296 PMC1828741

[19] Wang L, Maji SK, Sawaya MR, Eisenberg D, Riek R. 2008. Bacterial inclusion bodies contain amyloid-like structure. PLoS Biol 6:e195. doi:10.1371/journal.pbio.0060195. 18684013 PMC2494559

[20] Fujiwara K, Toda H, Ikeguchi M. 2012. Dependence of alpha-helical and beta-sheet amino acid propensities on the overall protein fold type. BMC Struct Biol 12:18. doi:10.1186/1472-6807-12-18. 22857400 PMC3495713

[21] Esposito D, Chatterjee DK. 2006. Enhancement of soluble protein expression through the use of fusion tags. Curr Opin Biotech 17:353–358. doi:10.1016/j.copbio.2006.06.003. 16781139

[22] Vidovic V, Prongidi-Fix L, Bechinger B, Werten S. 2009. Production and isotope labeling of antimicrobial peptides in *Escherichia coli* by means of a novel fusion partner that enables high-yield insoluble expression and fast purification. J Pept Sci 15:278–284. doi:10.1002/psc.1112. 19189273

[23] Zasloff M. 1987. Magainins, a class of antimicrobial peptides from Xenopus skin: isolation, characterization of two active forms, and 507 partial cDNA sequence of a precursor. Proc Natl Acad Sci USA 84:5449–5453. doi:10.1073/pnas.84.15.5449. 3299384 PMC298875

[24] Samakovlis C, Kylsten P, Kimbrell DA, Engstrom A, Hultmark D. 1991. The andropin gene and its product, a male-specific antibacterial peptide in Drosophila melanogaster. EMBO J 10:163–169. doi:10.1002/j.1460-2075.1991.tb07932.x. 1899226 PMC452624

[25] Levashina EA, Ohresser S, Bulet P, Reichhart JM, Hetru C, Hoffmann JA. 1995. Metchnikowin, a novel immune-inducible proline-rich peptide from Drosophila with antibacterial and antifungal properties. Eur J Biochem 233:694–700. doi:10.1111/j.1432-1033.1995.694_2.x. 7588819

[26] Nerelius C, Fitzen M, Johansson J. 2010. Amino acid sequence determinants and molecular chaperones in amyloid fibril formation. Biochem Biophys Res Commun 396:2–6. doi:10.1016/j.bbrc.2010.02.105. 20494101

[27] Singh A, Upadhyay V, Upadhyay AK, Singh SM, Panda AK. 2015. Protein recovery from inclusion bodies of *Escherichia coli* using mild solubilization process. Microb Cell Fact 14:41. doi:10.1186/s12934-015-0222-8. 25889252 PMC4379949

[28] Almeida ZL, Brito RMM. 2020. Structure and aggregation mechanisms in amyloids. Molecules 25:1195. doi:10.3390/molecules25051195. 32155822 PMC7179426

[29] de Groot NS, Aviles FX, Vendrell J, Ventura S. 2006. Mutagenesis of the central hydrophobic cluster in Abeta42 Alzheimer's peptide. Side-chain properties correlate with aggregation propensities. FEBS J 273:658–668. doi:10.1111/j.1742-4658.2005.05102.x. 16420488

[30] Morimoto A, Irie K, Murakami K, Masuda Y, Ohigashi H, Nagao M, Fukuda H, Shimizu T, Shirasawa T. 2004. Analysis of the secondary structure of beta-amyloid (Abeta42) fibrils by systematic proline replacement. J Biol Chem 279:52781–52788. doi:10.1074/jbc.M406262200. 15459202

[31] Beerten J, Jonckheere W, Rudyak S, Xu J, Wilkinson H, De Smet F, Schymkowitz J, Rousseau F. 2012. Aggregation gatekeepers modulate protein homeostasis of aggregating sequences and affect bacterial fitness. Protein Eng Des Sel 25:357–366. doi:10.1093/protein/gzs031. 22706763

[32] Aguilera P, Marcoleta A, Lobos-Ruiz P, Arranz R, Valpuesta JM, Monasterio O, Lagos R. 2016. Identification of key amino acid residues modulating intracellular and in vitro Microcin E492 amyloid formation. Front Microbiol 7:35. doi:10.3389/fmicb.2016.00035. 26858708 PMC4729943

[33] Hwang PM, Pan JS, Sykes BD. 2012. A PagP fusion protein system for the expression of intrinsically disordered proteins in Escherichia coli. Protein Expr Purif 85:148–151. doi:10.1016/j.pep.2012.07.007. 22841980

[34] Nguyen LT, Haney EF, Vogel HJ. 2011. The expanding scope of antimicrobial peptide structures and their modes of action. Trends Biotechnol 29:464–472. doi:10.1016/j.tibtech.2011.05.001. 21680034

[35] Li Y. 2011. Recombinant production of antimicrobial peptides in *Escherichia coli*: a review. Protein Expr Purif 80:260–267. doi:10.1016/j.pep.2011.08.001. 21843642

[36] Waugh DS. 2011. An overview of enzymatic reagents for the removal of affinity tags. Protein Expr Purif 80:283–293. doi:10.1016/j.pep.2011.08.005. 21871965 PMC3195948

[37] Zahran S, Pan JS, Liu PB, Hwang PM. 2015. Combining a PagP fusion protein system with nickel ion-catalyzed cleavage to produce intrinsically disordered proteins in *Escherichia coli*. Protein Expr Purif 116:133–138. doi:10.1016/j.pep.2015.08.018. 26297994

[38] Horton RM, Hunt HD, Ho SN, Pullen JK, Pease LR. 1989. Engineering hybrid genes without the use of restriction enzymes: gene splicing by overlap extension. Gene 77:61–68. doi:10.1016/0378-1119(89)90359-4. 2744488

[39] Protas AM, Ariani HHN, Bonna A, Polkowska-Nowakowska A, Poznański J, Bal W. 2013. Sequence-specific Ni (II)-dependent peptide bond hydrolysis for protein engineering: active sequence optimization. J Inorg Biochem 127:99–106. doi:10.1016/j.jinorgbio.2013.07.037. 23973681

[40] Sun CS, Liang JQ, Shi R, Gao XN, Zhang RJ, Hong FL, Yuan QH, Wang SB. 2012. Tobacco etch virus protease retains its activity in various buffers and in the presence of diverse additives. Protein Expr Purif 82:226–231. doi:10.1016/j.pep.2012.01.005. 22285121

[41] Chen N, Hong FL, Wang HH, Yuan QH, Ma WY, Gao XN, Shi R, Zhang RJ, Sun CS, Wang SB. 2012. Modified recombinant proteins can be exported via the Sec pathway in *Escherichia coli*. PLoS One 7:e42519. doi:10.1371/journal.pone.0042519. 22912705 PMC3418276

[42] Tian ZG, Teng D, Yang YL, Luo J, Feng XJ, Fan Y, Zhang F, Wang JH. 2007. Multimerization and fusion expression of bovine lactoferricin derivative LfcinB15-W4,10 in *Escherichia coli*. Appl Microbiol Biotechnol 75:117–124. doi:10.1007/s00253-006-0806-7. 17225098

