# Supplementary material for: Refinement of the Fusion Tag PagP for Effective Formation of Inclusion Bodies in Escherichia coli
Source: Microbiol Spectr. 2023 May 24;11(3):e03803-22. doi: 10.1128/spectrum.03803-22 (PMC10269538; doi:10.1128/spectrum.03803-22)
Supplement: Supplemental file 1 — Table S1. Download spectrum.03803-22-s0001.docx, DOCX file, 0.01 MB [file spectrum.03803-22-s0001.docx]

Table S1:

Primer sequences:

| Primer No. | Sequence (from 5' end to 3' end) |
| --- | --- |
| Primer 1  Primer 2  Primer 3  Primer 4  Primer 5  Primer 6  Primer 7  Primer 8  Primer 9  Primer 10  Primer 11  Primer 12  Primer 13  Primer 14  Primer 15  Primer 16  Primer 17  Primer 18  Primer 19  Primer 20  Primer 21  Primer 22  Primer 23  Primer 24  Primer 25  Primer 26  Primer 27  Primer 28  Primer 29  Primer 30  Primer 31  Primer 32 | GAAAACCTGTACTTCCAGCACCGTCACCAGGGTCCGAT  CGGAGACGGACGGGTGTCGAAGATCGGACCCTGGTGACGG  GACACCCGTCCGTCTCCGTTCAACCCGAACCAGCCGCGTC  ATCAAGCTTTTAGTAGATCGGACCCGGACGCGGCTGGTTCGGG  GAAAACCTGTACTTCCAGGGTATCGGTAAATTCCTGCACAGCGCT  TTCACCCACGAATGCTTTACCGAATTTCTTAGCGCTGTGCAGGAA  GCTAAGCTTTTAAGAGTTCATGATTTCACCCACGAATGC  GGTCATCACCATCACCATCATGGTGAAAACCTGTACTTCCAG  GTCATTGGATGGCTGGTGGTCATCACCATCACCATC  TCCGAATTCGCATCTCGTCATTGGATGGCTGGTG  GAAAACCTGTACTTCCAGGTTTTCATCGACATCCTGGACAAAGTT  AACCTGCGCCGCGTTGTGGATCGCGTTTTCAACTTTGTCCAGGAT  AACGCGGCGCAGGTTGGTATCGGTTTCGCGAAACCGTTCGAAAAA  TTTCGGGTTGATCAGTTTTTCGAACGGTTTCGCGAAACCGATA  GCTAAGCTTATTTCGGGTTGATCAGTTTTTC  ATTAGCATATGAACGCAGATGAGTGGATGAC  ATGGATCCAAACTGAAAGCGCATCCAGG  CGTCCATGGGTAATTTTCATTTAGGTCTGG  CGTCCATGGGTAACGCAGATGAGTGGAATTTTCATTTAGGTCTGG  GTGGATGACAACGTTTAGAAATTTTCATTTAGGTCTGG  CGTCCATGGGTAACGCAGATGAGTGGATGACAACGTTTAG  CGTCCATGGGTCATGGCCTGTATGCCATGGCAAATTTTCATTTAGGTCTG  CGTCCATGGGCGGTTTTGGCCTGTCGCATGGCCTGTATGC  CGTCCATGGGTGCCATCACCTGGCATGCACGTGGCGGTTTTGGCCTGTCG  ATTAGCATATGAGCGATAAAATTATTCACC  ATGGATCCCGCCAGGTTAGCGTCGAGG  GATAACTGGAATTACATCCTTCTCCTGGTTCTACTGCCATTGGCC  GGCCAATGGCAGTAGAACCAGGAGAAGGATGTAATTCCAGTTATC  CCCTCTCCCGGTTCTACTGATATTGGCCTCCGTGGGTTATG  CATAACCCACGGAGGCCAATATCAGTAGAACCGGGAGAGGG  CCTCCGTGGGTTATGGCCTAGTGACTTTTCAGATGAC  GTCATCTGAAAAGTCACTAGGCCATAACCCACGGAGG |

Nucleotide sequence of the histone fold domain (HFD) of human transcription factor TAF12 (HFD-TAF) gene:

ATGGTTCTGACCAAAAAGAAACTGCAGGACCTGGTTCGTGAAGTTGCGCCGAACGAACAGCTGGACGAAGACGTTGAAGAAATGCTGCTGCAGATCGCGGACGACTTCATCGAATCTGTTGTTACCGCGGCGTGCCAGCTGGCGCGTCACCGTAAATCTTCTACCCTGGAAGTTAAAGACGTTCAGCTGCACCTGGAACGTCAGTGGAACATGTGGATC
