# Supplementary material for: Refinement of the Fusion Tag PagP for Effective Formation of Inclusion Bodies in Escherichia coli
Source: Microbiol Spectr. 2023 May 24;11(3):e03803-22. doi: 10.1128/spectrum.03803-22 (PMC10269538; doi:10.1128/spectrum.03803-22)
Supplement: Supplemental file 2 — Table S2. Download spectrum.03803-22-s0002.docx, DOCX file, 0.01 MB [file spectrum.03803-22-s0002.docx]

Table S2:

Amino acid sequences of fusion tags and antimicrobial peptides:

| Fusion tags or antimicrobial peptides | Amino acid sequences |
| --- | --- |
| PagP  PagP-1  PagP-2  PagP-3  PagP-4  PagP-5  PagP-6  TrxA  HFD-TAF12  NHT  Magainin II | NADEWMTTFRENIAQTWQQPEHYDLYIPAITWHARFAYDKEKTDRYNERPWGGGFGLSRWDEKGNWHGLYAMAFKDSWNKWEPIAGYGWESTWRPLADENFHLGLGFTAGVTARDNWNYIPLPVLLPLASVGYGPVTFQMTYIPGTYNNGNVYFAWMRFQF  NFHLGLGFTAGVTARDNWNYIPLPVLLPLASVGYGPVTFQMTYIPGTYNNGNVYFAWMRFQF  NADEWNFHLGLGFTAGVTARDNWNYIPLPVLLPLASVGYGPVTFQMTYIPGTYNNGNVYFAWMRFQF  NADEWMTTFRNFHLGLGFTAGVTARDNWNYIPLPVLLPLASVGYGPVTFQMTYIPGTYNNGNVYFAWMRFQF  HGLYAMANFHLGLGFTAGVTARDNWNYIPLPVLLPLASVGYGPVTFQMTYIPGTYNNGNVYFAWMRFQF  GGFGLSHGLYAMANFHLGLGFTAGVTARDNWNYIPLPVLLPLASVGYGPVTFQMTYIPGTYNNGNVYFAWMRFQF  AITWHARGGFGLSHGLYAMANFHLGLGFTAGVTARDNWNYIPLPVLLPLASVGYGPVTFQMTYIPGTYNNGNVYFAWMRFQF  SDKIIHLTDDSFDTDVLKADGAILVDFWAEWCGPCKMIAPILDEIADEYQGKLTVAKLNIDQNPGTAPKYGIRGIPTLLLFKNGEVAATKVGALSKGQLKEFLDANLA  VLTKKKLQDLVREVAPNEQLDEDVEEMLLQIADDFIESVVTAACQLARHRKSSTLEVKDVQLHLERQWNMWI  ASRHWMAGGHHHHHHGENLYFQ  GIGKFLHSAKKFGKAFVGEIMNS |
| Metchnikowin | HRHQGPIFDTRPSPFNPNQPRPGPIY |
| Andropin | VFIDILDKMENAIHKAAQAGIGIAKPIEKMILPK |
